# *MSH6* missense mutations are often associated with no or low cancer susceptibility

**DOI:** 10.1038/sj.bjc.6602129

**Published:** 2004-08-31

**Authors:** R Kariola, H Hampel, W L Frankel, T E Raevaara, A de la Chapelle, M Nyström-Lahti

**Affiliations:** 1 Department of Biological and Environmental Sciences, Genetics, University of Helsinki, FI-00014 Helsinki, Finland; 2 Human Cancer Genetics Program, Comprehensive Cancer Center, The Ohio State University, Columbus, OH 43210, USA; 3 Department of Pathology, The Ohio State University, Columbus, OH 43210, USA

**Keywords:** functional analysis, HNPCC, mismatch repair, MSH6

## Abstract

Mismatch repair (MMR) deficiency in tumours from patients with the hereditary nonpolyposis colorectal cancer (HNPCC) syndrome is mainly caused by mutations in the *MLH1, MSH2*, and *MSH6* genes. A major challenge in the clinical management of patients with suspected HNPCC is the frequent occurrence of missense mutations in *MSH6*. These can be considered neither deleterious nor clinically innocent *a priori*. To assess their significance we studied five novel *MSH6* missense mutations in six patients derived from a series of consecutive endometrial and colorectal cancer patients selected for study after their tumours were determined to be microsatellite unstable. We tested each mutated protein for heterodimerisation with MSH2 and for *in vitro* MMR capability. Four mutations (R128L, P623L, K728T, G881K+S) showed no impairment of these functions while the fifth (E1193K) displayed marked impairment of both functions. These results, taken together with our previous similar findings concerning six other missense mutations in *MSH6*, allow us to conclude that many or most missense changes in *MSH6* likely are clinically innocent, whereas some missense changes such as E1193K, which lead to impaired MMR, are likely to be clinically significant, but have low penetrance.

The cancer susceptibility in hereditary nonpolyposis colorectal cancer (HNPCC) syndrome is associated with defective DNA mismatch repair (MMR). An inherited defect in one MMR allele and an acquired defect in the other allele lead to loss of MMR function in the respective polypeptide that accelerates tumour progression. Accordingly, lack of the mutated protein and instability at short tandem repeat sequences such as microsatellites in the genome are the main molecular characteristics of the HNPCC tumours.

More than 450 different MMR gene mutations and 100 intragenic polymorphisms, affecting mostly the MMR genes, *MLH1, MSH2*, and *MSH6*, are listed in the international HNPCC mutation database (http://www.nfdht.nl). The clinical phenotype varies between families. Contrary to most mutations affecting the *MLH1* and *MSH2* genes, a significant proportion of *MSH6* mutations occur in HNPCC families with less typical clinical features. Carriers of *MSH6* mutations often display late age at onset, carcinomas of the endometrium, and low or no microsatellite instability (MSI) in tumours (Miyaki *et al*, 1997; Kolodner *et al*, 1999; Wijnen *et al*, 1999; Wu *et al*, 1999; Parc *et al*, 2000; Wagner *et al*, 2001; Berends *et al*, 2002; Peterlongo *et al*, 2003; Cederquist *et al*, 2004). Moreover, one-third of *MSH6* mutations are nontruncating, and so do not necessarily destroy the encoded protein. These mutations may be associated with a positive immunohistochemical (IHC) staining for the MSH6 protein, which makes them even more difficult to interpret. In the mutation database, 12 *MSH6* amino-acid substitutions are listed as pathogenic mutations, while 16 nucleotide changes in the *MSH6* coding sequence are listed as polymorphisms. In both groups some changes are found to segregate in putative HNPCC families, while others occur in single endometrial cancer (EC) or colorectal cancer (CRC) patients, which impedes segregation analysis and makes their interpretation impossible.

To address the question, which, if any, *MSH6* missense mutations cause susceptibility to HNPCC, we previously studied the functionality of six MSH6 variants (S144I, G566R, P1087T, P1087R, R1095H, and L1354Q) (Kariola *et al*, 2002, 2003). Here, we expanded the study by analysing five novel *MSH6* missense mutations (R128L, P623L, K728T, G881K+S, and E1193K). Distinct from the previous cases, these mutations were derived from a population-based study of EC and CRC patients whose tumours showed MSI, the main hallmark of HNPCC. Our experimental results in conjunction with the clinical characteristics of the patients provide evidence that most *MSH6* missense mutations found in a population-based case series of patients with MSI positive tumours are unlikely to be deleterious.

## MATERIALS AND METHODS

### Patient specimens

*MSH6* missense mutations were derived from a project comprising altogether 2000 colorectal and EC cases newly diagnosed in the main hospitals of the Columbus metropolitan area in central Ohio (population 1.5 million). In total, 19% of colorectal and 22% of endometrial tumours showed instability with at least one of five microsatellite markers (Boland *et al*, 1998). Blood DNA was studied for mutations in all patients with microsatellite unstable tumours. The six patients chosen for this study were the ones, in which a novel *MSH6* missense mutation in the coding region was detected, no other mutations detected by DNA sequencing occurred in *MSH6, MLH1*, or *MSH2*, and the clinical significance of the change could not be evaluated based on available evidence. All studies were approved by the Institutional Review Boards of the Ohio State University and the University of Helsinki.

### Microsatellite instability analysis

Tumours were microdissected as described previously (Chadwick *et al*, 2001) and DNA extracted from tissue that contained at least 50% tumour cells. Unaffected DNA was obtained either from a blood sample or from microdissected endometrial tissue containing no tumour cells. For the analysis, a panel of five polymorphic microsatellite markers *(BAT25, BAT26, D2S123, D5S346*, and *D17S25* or *D18S69*) was used.

### Mutation detection

Direct exon-by-exon sequencing of *MLH1, MSH2*, and *MSH6* was carried out in an ABI automated sequencer as described (Chadwick *et al*, 2001). In *MSH6*, all the exons with the exception of one part of exon 4 were sequenced in a single PCR reaction (Wu *et al*, 1999).

### Population frequency of missense changes

All five missense changes were searched for by SSCP using primers and conditions available from the authors. All SSCP variants were sequenced. The control population consisted of 140 individuals of mixed European ethnicity including 40 grandparents from the Centre d'Etude du Polymorphisme Humain collection obtained from the Coriell Institute, New Jersey.

### Immunohistochemical analysis

Paraffin embedded tissue was cut into 4 *μ*M sections and placed on positively charged slides. The slides were placed in a 60°C oven for 1 h, cooled, and deparaffinised and rehydrated through xylenes and graded ethanol solutions to water. All slides were quenched for 5 min in a 3% hydrogen peroxide solution in methanol to block for endogenous peroxidase. Antigen retrieval was performed by a heat method for all three antibodies, in which the specimens were placed in a citric acid solution (Dako's Target Retrieval Solution, pH 6.1), for 30 min at 94°C using a vegetable steamer. After allowing slides to cool for 15 min at room temperature, slides were placed on a Dako Autostainer immunostaining system, for use with immunhistochemistry. The primary antibody was incubated for 1 h at room temperature. The primary antibodies used were MLH1, clone G168-728 (BD PharMingen) 1/60; MSH2, Ab-2 (Oncogene Research Products) 1/200; and MSH6 (Transduction Laboratories) 1/400. The detection system used for all antibodies was a labelled Streptavidin–Biotin complex. This method is based on the consecutive application of (1) a primary antibody against the antigen to be localised, (2) biotinylated linking antibody, (3) enzyme conjugated streptavidin, and (4) substrate chromogen (DAB). The tissue was protein blocked using Dako's serum free protein block prior to the primary antibody application. Endogenous avidin and biotin were blocked prior to the biotinylated-linking antibody. Slides were then counterstained in Richard Allen hematoxylin, dehydrated through graded ethanol solutions and coverslipped.

### *MLH1* methylation analysis

Two sections of the *MLH1* promoter region were studied for methylation. Methylation-specific PCR was used to assess a region approximately 700 bp upstream of the translation initiation site (Herman *et al*, 1998), designated H in this report. A combination of bisulphite PCR and cleavage by restriction enzyme digestion was used to study a second region that lies immediately upstream of the translational start site (Deng *et al*, 1999) and designated D in this report. The methods have been described in detail previously (Nakagawa *et al*, 2001).

### Site-directed mutagenesis and production of recombinant proteins in insect cells

Site-directed mutagenesis was used to introduce the mutations into *MSH6* cDNA, which was cloned into the plasmid pFastBac1 (Gibco BRL), as described previously (Kariola *et al*, 2002, 2003). The primer sequences, PCR product sizes, and cloning sites are listed in Table 2

**Table 2:** Experimental conditions for the site-directed mutagenesis

**Mutation**	**Oligos for fragments A and B in 1st PCR (5′ → 3′) (the mutated site is marked with bold letter)**	**Size (bp)**	**Oligos for 2nd PCR**	**Size (bp)**	**Cloning site**
MSH6-R128L	FA: CGGATTATTCATACCGTCCC	435	FA	1282	*BamHI*
	RA: CTGTACATGAACA**A**GGACTGATTTCCC				*Eco81I*
	FB: GGGAAATCAGTCC**T**TGTTCATGTACAG	874	RB		
	RB: GCCACCACTTCCTCATCC				
MSH6-P623L	FA: GATGAGCACAGGAGGAGGC	746	FA	1599	*Eco81I*
	RA: CAAAACTGGGAGCCG**A**GTATCAGACCTTC				*PstI*
	FB: GAAGGTCTGATAC**T**CGGCTCCCAGTTTTG	882	RB		
	RB: CCATCGGTTCAATTCTACAGTC				
MSH6-K728T	FA: GATGAGCACAGGAGGAGGC	1058	FA	1599	*Eco81I*
	RA: CGTTGATAGGCT**G**TGGTGAAGATAGC				*PstI*
	FB: GCTATCTTCACCA**C**AGCCTATCAACG	567	RB		
	RB: CCATCGGTTCAATTCTACAGTC				
MSH6-G881K+S	FA: GATGAGCACAGGAGGAGGC	1518	FA	1602	*Eco81I*
	RA: AGACTTAAAAC**TTTT**ATCAGCAACTTC				*PstI*
	FB: GAAGTTGCTGAT**AAAA**GTTTTAAGTCT	111	RB		
	RB: CCATCGGTTCAATTCTACAGTC				
MSH6-E1193K	FA: GTCTAAAATCCTTAAGCAGG	942	FA	1612	*PstI*
	RA: GTTTCACTTAATT**T**AACAAAAAATGTAC				*XhoI*
	FB: GTACATTTTTTGTT**A**AATTAAGTGAAAC	698	RB		
	RB: CTGATTATGATCCTCTAGTAC				

. The mutated cloned fragments were verified by DNA sequencing (AbiPrism 3100 Genetic Analyzer, Applied Biosystems). The recombinant baculoviruses, which were used as expression vectors, were generated using the Bac-to-Bac system according to the manufactureŕs instructions (Gibco BRL). For protein production, *Sf9* insect cells were co-transfected with *MSH2* and *MSH6* recombinant baculoviruses in combinations of wild-type *MSH2* (*MSH2*-WT) with mutated *MSH6* (*MSH6*-R128L, *MSH6*-P623L, *MSH6*-K728T, *MSH6*-G881K+S, or *MSH6*-E1193K) or with wild-type *MSH6* (*MSH6-*WT). The co-transfection was used since both *in vivo* studies in mice (de Wind *et al*, 1999) and *in vitro* studies in human cells (Marra *et al*, 1998; Chang *et al*, 2000) have shown that the MSH6 protein (Msh6 in the mouse) is unstable in the absence of its partner MSH2 (Msh2). After 72 h of culture, the total protein extracts (TE), including the overexpressed MSH6 and MSH2 proteins, were prepared as described previously (Nyström-Lahti *et al*, 2002).

### Combined co-immunoprecipitation and Western blot analysis

The combined co-immunoprecipitation and Western blot analysis was performed according to previous reports (Kariola *et al*, 2002, 2003). The total protein extracts, which were estimated to contain equal quantities of recombinant wild-type MSH2 protein and the immunoprecipitated samples obtained with anti-MSH6 monoclonal antibody (clone 44, BD Transduction Laboratories), were loaded on 7.5% SDS–polyacrylamide gels. After electrophoresis, the proteins were transferred onto nitrocellulose membranes (Hybond-P PVDF, Amercham Pharmacia Biotech), which were blotted with monoclonal antibodies against MSH6 (0.02 *μ*g ml^−1^, clone 44, BD Transduction Laboratories) and MSH2 (0.3 *μ*g ml^−1^, Ab-1, Oncogene Research Products).

### *In vitro* MMR assay

The functionality of the recombinant MutS*α* (MSH2/MSH6) variants was studied in the *in vitro* MMR assay (Kariola *et al*, 2002, 2003). In the assay, circular DNA heteroduplexes, which contain a G•T mispair 369 base pairs downstream from a single-strand nick were used as substrates. The functionality of the mutated MSH6 proteins was studied by complementing MMR-deficient nuclear extract (NE) of HCT15 (MSH6^−/−^) cells with the total protein extract (TE) of *Sf9* cells including over expressed human MSH6 and MSH2 proteins. Each protein extract was estimated to contain equal quantities of recombinant wild-type MSH2. NE of the MMR-proficient cell line TK6, and HCT15 complemented with wild-type MutS*α* TE, were used as positive controls and HCT15 NE as a negative control in the assay. If the repair reaction occurs, it converts G•T heteroduplex to A•T homoduplex, whereafter restriction enzyme *Bgl*II can cleave the molecule and the repair efficiency can be measured by the cleavage efficiency. The quantification of the repaired DNA was carried out with Image-Pro Version 4.0 (MediaCybernetics®). Since the heteroduplex molecules are not all repairable they are always added in excess in the assay, and the repair capacity of the proficient wild-type controls is used as a reference level.

## RESULTS

### Genetic and clinical data in *MSH6* mutation carriers

Five novel *MSH6* missense mutations (MSH6-R128L, MSH6-P623L, MSH6-K728T, MSH6-G881K+S, MSH6-E1193K) were found in six patients, whose endometrial or colorectal tumour showed high MSI (when at least two out of five markers showed instability). In each case, mutations detectable by DNA sequencing had been excluded in *MLH1* and *MSH2*. Furthermore, the missense changes had not been found in 140 control individuals of comparable ethnicity. The ages at diagnosis were high, with a range of 58–83 years, and none of the mutation carriers had first-degree relatives with EC or CRC. The identified mutations and molecular and clinical data are shown in Table 1

**Table 1:** Genetic and clinical data of families with *MSH6* germline missense mutations

								**MSI^b^**							**Immunohistochemistry**			
**Case**	**Exon**	**Codon**	**Nucleotide change**	**Predicted coding change**	**Conserved codon^a^**	**Frequency in controls**	**Proband's tumour (age, year)**	**BAT25**	**BAT26**	**D2S123**	**D5S346**	**D17S25**	**D18S69**	**MLH1 promoter methylation**	**MLH1**	**MSH2**	**MSH6**	**Relatives with cancer**
313	2	128	CGT → CTT at 383	Arg → Leu	No	0/140	EC (68)	−	−	+	−	ND	+	H+D+^c^	−	+	+	None
266	4	623	CCC → CTC at 1868	Pro → Leu	No	0/140	EC (58)	+	+	−	+	+	ND	H+D+	+	+	−	Paternal aunt CRC (63)
371	4	728	AAA → ACA at 2183	Lys → Thr	Yes	0/140	CRC (82)	+	+	+	+	+	ND	H+D+	−	+	+	Daughter breast cancer (30)
509	4	881	GGT → AAAAGT at 2641	Gly → Lys +Ser	No	0/140	EC (83)	−	+	+	−	+	ND	H+D+	−	+	+	Maternal half brother melanoma
501	7	1193	GAA → AAA at 3577	Glu → Lys	Yes	0/140	EC (60)	+	+	−	−	+	ND	H−D−	+	−	−	Brother skin cancer (60)
553	7	1193	GAA → AAA at 3577	Glu → Lys	Yes	0/140	EC (59)	+	−	+	−	ND	−	H−D−	+	+	5%	None

a Conserved through human, yeast and mouse.

b MSI, microsatellite instability; BAT25 and BAT26 are mononucleotide markers; D2S123, D5S346, D17S25, and D18S69 are dinucleotide markers; ND, not determined.

c H and D refer to different parts of the promoter region described in Materials and Methods. + denotes methylation, – denotes no methylation.

.

IHC staining for MLH1, MSH2, and MSH6 revealed that MSH6 protein was lost or nearly lost (5%) in three ECs, derived from the patient carrying the mutation P623L and the two women both carrying the mutation E1193K. One of the latter tumours also lacked MSH2 protein, while MLH1 was expressed. MLH1 protein was absent in three tumours (R128L, K728T, G881K+S), in which MSH2 and MSH6 were expressed. The lack of MLH1 in these tumours was consistent with the finding that the same tumours also showed hypermethylation of the *MLH1* promoter region, which often leads to *MLH1* gene inactivation and loss of MMR function.

### Interactions of the MSH6 variants with wild-type MSH2

**Figure 1:**
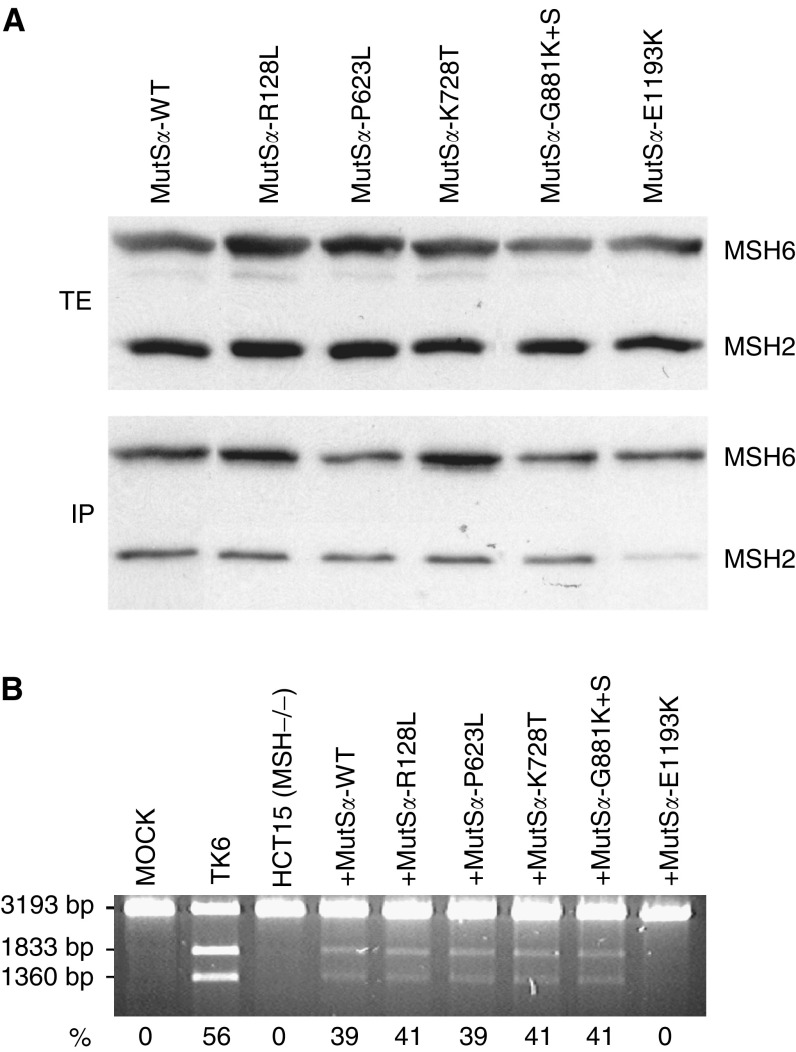
(**A**) Combined immunoprecipitation and Western blot analysis showing interactions of MSH6 variants with wild-type MSH2. The upper panel shows a Western blot including total protein extracts (TE), the lower panel shows immunoprecipitates (IP) obtained with anti-MSH6 antibody. Following transfer onto the membrane, the proteins were visualised with anti-MSH6 and anti-MSH2 antibodies (see Materials and Methods). The figure shows that the mutated MSH6 proteins R128L, P623L, K728T, and G881K+S are able to form stable MSH2-MSH6 heterodimers and co-precipitate a similar amount of the MSH2 protein as wild-type MSH6, while MSH6-E1193K co-precipitates clearly less MSH2 protein. (**B**) MMR activity of MMR deficient nuclear extract of HCT15 cells complemented with wild-type MutS*α* (MutS*α*-WT) and mutated variants. Mock contains heteroduplex DNA with no protein, TK6 is an MMR-proficient and HCT15 an MMR-deficient (MSH6^−/−^) control (see Materials and Methods). The fragment lengths on the left indicate the migration of the unrepaired linearised plasmid DNA (3193 bp) and of the two fragments (1833 and 1360 bp) produced following correction of the G•T mispair, which makes the DNA susceptible to cleavage with the restriction endonuclease *Bgl*II. The numbers below the panel represent fractions (%) of repaired DNA and display absolute MMR deficiency (0%) of MutS*α*-E1193K. The values are an average of two independent experiments.

Combined co-immunoprecipitation and Western blot analysis was used to study the effect of *MSH6* mutations on the heterodimerisation of MSH6 and MSH2 proteins. Wild-type MutS*α* (MutS*α*-WT) was used as a control. As shown in Figure 1A

, the total protein extracts from *Sf9* cells used for immunoprecipitation assay contained similar amounts of wild-type MSH2 (MutS*α*-WT, MutS*α*-R128L, MutS*α*-P623L, MutS*α*-K728T, MutS*α*-G881K+S, and MutS*α*-E1193K). The immunoprecipitates obtained with anti-MSH6 antibody show that co-expression of four of the MSH6 variants (MSH6-R128L, MSH6-P623L, MSH6-K728T, and MSH6-G881K+S) with wild-type MSH2 yielded stable heterodimers, since the mutated MSH6 co-precipitated similar amount of MSH2 as wild-type MSH6. In contrast, MSH6-E1193K co-precipitated much less MSH2 than MSH6-WT, suggesting interference in their heterodimerisation.

### Mismatch repair capacity of MutS*α* heterodimers

The functionality of the MutS*α* variants was tested in an *in vitro* MMR assay (Figure 1B). MutS*α*-WT, MutS*α*-R128L, MutS*α*-P623L, MutS*α*-K728T, and MutS*α*-G881K+S were able to complement the MSH6-deficient NE of HCT15 cells and repaired 39, 41, 39, 41, and 41% of G•T heteroduplexes, respectively. However, MutS*α*-E1193K protein complex failed to complement HCT15 NE and displayed complete MMR deficiency (0%).

## DISCUSSION

Genetic counselling of mutation carriers must be based on a reliable assessment of the pathogenicity of the mutation. The present study was undertaken to evaluate the pathogenicity of inherited missense alterations in the MMR gene *MSH6*. To date, *MSH6* gene is the third most common susceptibility gene in the HNPCC syndrome accounting for about 10% of reported mutations found in HNPCC kindreds (http://www.nfdht.nl). Missense mutations, which account for 38% of all *MSH6* mutations, are particularly challenging in the clinical management of patients with suspected HNPCC. Even when the amino-acid substitution is pathogenic, the mutation does not necessarily lead to the absence of the protein; thus, it can be detectable by IHC analysis. Furthermore, segregation studies to distinguish pathogenic mutations from polymorphisms are frequently hindered by small family size.

We have previously studied the functionality of six MSH6 variants (S144I, G566R, P1087T, P1087R, R1095H, and L1354Q), four of which are listed as pathogenic mutations in the mutation database. The mutations were found in HNPCC kindreds, which did not fulfil the Amsterdam criteria (I or II) and were classified as non-HNPCC or suspected HNPCC families (Vasen *et al*, 1991, 1999). The same functional assay was previously used to detect impaired MMR caused by the *MLH1* missense mutations C77R, I107R, and R659P (Nyström-Lahti *et al*, 2002). Interestingly, all the six mutated MSH6 proteins were able to repair mismatches (Kariola *et al*, 2002, 2003), suggesting no MMR defect detectable in our assay. To further address the question of whether *MSH6* missense mutations cause MMR defect and susceptibility to HNPCC, the pathogenicity of five novel *MSH6* missense variations (R128L, P623L, K728T, G881K+S, and E1193K) was evaluated in the present study. These changes were derived from a series of consecutive EC and CRC patients selected for study after their tumours were determined to be microsatellite unstable. Importantly, in each case, the mutation was searched for in 140 control individuals and not found. Thus, these changes could not be classified *a priori* as likely polymorphisms. Irrespective of these facts, none of the mutation carriers was shown to have first-degree relatives with EC or CRC or any family history of cancer suggestive of HNPCC.

We studied the interactions of five mutated MSH6 proteins with normal MSH2 and tested the functionality of these heterodimers of MSH6 and MSH2 in the *in vitro* MMR assay. Four mutations (R128L, P623L, K728t, G881K+S) showed no impairment of these functions while the fifth mutation (E1193K) displayed marked impairment of both functions. The MSH6-E1193K protein co-precipitated much less MSH2 than wild-type MSH6, and the heterodimer MutS*α*-E1193K could not repair mismatches. The E1193K mutation occurred in two independently ascertained women with EC (ages at onset 59 and 60 years). Both tumours stained negatively or very weakly for MSH6 protein, which is compatible with MMR deficiency associated with MSH6 mutation. The amino acid E1193 is located in a highly conserved region between the ATP and Mg^2+^ binding sites in *MSH6* (Iaccarino *et al*, 1998, 2000; Dufner *et al*, 2000) and, accordingly, can be expected to be sensitive to alterations. Irrespective of the definite MMR deficiency associated with the E1193K mutation, the families of the two patients, their siblings, excluding one brother diagnosed with skin cancer at 60, parents and children were unaffected. In immunohistochemical analysis, the three tumours derived from patients carrying mutations R128L, K728T, and G881K+S, expressed MSH6 but lacked MLH1 protein, which may explain the MSI phenotype in these tumours and suggests IHC staining for the MLH1 protein as the first approach in microsatellite unstable tumours. However, the mutation, P623L, which had no detectable phenotype in our functional assay was found in a patient, whose EC showed high MSI, and MSH6 was the only nonexpressed protein in the tumour among the three proteins tested. Thus, we cannot exclude the possibility that pathogenicity of P623L is caused by lower levels of the functional MSH6 protein as we have previously shown to be the case with some *MLH1* mutations (Raevaara *et al*, 2003). Another possibility is that *MSH6* inactivation is not at all due to P623L but has been acquired as a somatic event during the development of the tumour.

Our results, taken together with our previous similar findings concerning six other missense mutations in *MSH6* and the clinical data of the patients and their families, lead us to conclude that most missense changes in *MSH6* cause no or low cancer susceptibility. The results do not exclude the possibility that some of the studied *MSH6* mutations, which were functional in the *in vitro* MMR assay, could still affect biochemical events preceding the MMR function *in vivo.* Yet, a functional MMR defect, especially in situations where cosegregation of missense mutation and disease phenotype cannot be studied, reliably confirms that cancer susceptibility in a family is linked to a found mutation.
